# Short-Term Effects of Hydrokinesiotherapy in Hospitalized Preterm Newborns

**DOI:** 10.1155/2016/9285056

**Published:** 2016-09-08

**Authors:** Welcy Cassiano de Oliveira Tobinaga, Cirlene de Lima Marinho, Vera Lucia Barros Abelenda, Paula Morisco de Sá, Agnaldo José Lopes

**Affiliations:** ^1^Department of Physical Therapy, Pedro Ernesto University Hospital, State University of Rio de Janeiro, Boulevard 28 de Setembro 77, 20551-030 Vila Isabel, RJ, Brazil; ^2^Postgraduate Program in Clinical and Experimental Physiopathology (FISCLINEX), School of Medical Sciences, State University of Rio de Janeiro, Avenida Professor Manuel de Abreu 444, 20550-170 Vila Isabel, RJ, Brazil; ^3^Rehabilitation Sciences Postgraduate Program, Augusto Motta University Center, Avenida Paris 72, 21041-020 Bonsucesso, RJ, Brazil

## Abstract

*Background*. In the neonatal intensive care unit (NICU) environment, preterm newborns are subject to environmental stress and numerous painful interventions. It is known that hydrokinesiotherapy promotes comfort and reduces stress because of the physiological properties of water.* Objective*. To evaluate the short-term effects of hydrokinesiotherapy on reducing stress in preterm newborns admitted to the NICU.* Materials and Methods*. Fifteen preterm newborns underwent salivary cortisol measurement, pain evaluation using the Neonatal Infant Pain Scale (NIPS), and heart rate, respiratory rate, and peripheral oxygen saturation measurements before and after the application of hydrokinesiotherapy.* Results*. The mean gestational age of the newborns was 34.2 ± 1.66 weeks, and the mean weight was 1823.3 ± 437.4 g. Immediately after application of hydrokinesiotherapy, a significant reduction was observed in salivary cortisol (*p* = 0.004), heart rate (*p* = 0.003), and respiratory rate (*p* = 0.004) and a significant increase was observed in peripheral oxygen saturation (*p* = 0.002). However, no significant difference was observed in the NIPS score (*p* > 0.05).* Conclusion*. In the present study, neonatal hydrotherapy promoted short-term relief from feelings of stress. Neonatal hydrokinesiotherapy may be a therapeutic alternative. However, this therapy needs to be studied in randomized, crossover, and blinded trials. This trial is registered with NCT02707731.

## 1. Introduction

The neonatal intensive care unit (NICU) in Brazil has experienced major technological breakthroughs in recent years, following the global trend. These breakthroughs have contributed to a considerable decrease in mortality rates, allowing preterm newborns, especially those with low birth weight, to survive [1]. Annually, 15 million preterm newborns are born worldwide. Among all countries, Brazil has the tenth highest number of premature births, with 279,000 such births per year, corresponding to 9.2% of live births. Low birth weight is associated with increased neonatal and childhood morbidity and mortality [1, 2].

Between the twentieth and the twenty-fourth weeks of gestation, the fetus is already able to sense painful stimuli. The neuronal synapses are complete, and free nerve endings have specific receptors for pain perception. Interestingly, infants may feel pain with even more intensity than children and adults because of their immature inhibitory control mechanisms, which hamper their ability to modulate the pain experience [3]. During their hospital stay in the NICU, preterm newborns are subject to numerous painful interventions necessary for their survival. Some researchers estimate that a newborn admitted to the NICU is submitted to between 50 and 150 painful bedside procedures daily [4]. In addition to the painful interventions, preterm infants are exposed to a variety of adverse environmental conditions, including excessive light, loud noises, and frequent handling. This exposure, when maximized, can cause elevations in circulating cortisol levels, metabolic, behavioral, and physiological changes in the brain microstructure, susceptibility to infection, and delayed neurodevelopment [5–8].

When a newborn is exposed to a stressful stimulus, the hypothalamic-pituitary-adrenal axis is activated and releases adrenocorticotropic hormone, which in turn stimulates glucocorticoid secretion by the adrenal gland cortex. At high levels, glucocorticoid is considered to be an indicator of physical and psychological stress [5, 6, 9–12]. In this context, salivary cortisol measurement is indicated for the neonatal population, as it is a simple, noninvasive, and safe procedure for evaluating the stress threshold of a newborn [11–14]. Cândia et al. [13] and Takahashi et al. [15] have found that measuring salivary cortisol levels is a useful tool for evaluating the stress response in preterm newborns [13, 15]. However, Maas et al. [16] noted that the sensitivity and specificity of salivary cortisol in the detection of suprarenal insufficiency in newborns are low, with values of 0.66 and 0.62, respectively.

Given the harmful consequences of an unhealthy environment such as the NICU, it is necessary to incorporate daily therapeutic measures, preferably noninvasive procedures, that promote comfort, minimize stress, and provide pain relief. In recent decades, efforts have been made in multidisciplinary programs regarding the introduction of new concepts related to humanization and care in newborn development [17]. Paying special attention to pain, stress, and discomfort as well as techniques intended to reduce them can provide a starting point for improved quality of life for preterm newborns hospitalized in neonatal units. Although hydrokinesiotherapy has been used as a therapeutic technique for thousands of years, it is still a therapy that is not well understood nor well accepted in newborn care, and there are few studies on this approach in the literature. Further investigation into the technique is therefore of the utmost importance [18–23]. Neonatal hydrokinesiotherapy is a therapeutic alternative that allows the newborn to make movements that are facilitated by the aquatic environment, encouraging bone resorption metabolism, pain relief, and relaxation [14, 18–23]. Although it is considered an appropriate technique because of its many benefits, no studies have evaluated the changes in salivary cortisol measurement caused by neonatal hydrokinesiotherapy.

As a method that is simple to execute, nonpharmacological, and of low cost, neonatal hydrokinesiotherapy appears to provide many benefits, including accelerating the growth and development of biological systems in newborns [3, 9, 18, 19, 23–25]. Given the potential benefits of the technique, the objective of the present study was to investigate the short-term effects of hydrokinesiotherapy on salivary cortisol values in preterm newborns and, secondarily, to evaluate the short-term effects of the technique on the hemodynamics, respiratory parameters, and pain levels of these preterm infants.

## 2. Methods

### 2.1. Participants

Between July 2015 and February 2016, we evaluated 29 preterm newborns hospitalized in the NICU of the Pedro Ernesto University Hospital of the State University of Rio de Janeiro, Brazil, who underwent hydrokinesiotherapy. The inclusion criteria consisted of preterm newborn infants with a gestational age of less than 37 weeks [1], birth weight > 1000 g, and clinical stability. The study excluded infants with intravascular cannulas (intravenous or arterial lines) below shoulder level, the presence of umbilical catheterization, the presence of umbilical cord remnants, neurological or heart problems, and the presence of orthopedic or facial malformations that made it impossible to apply the Neonatal Infant Pain Scale (NIPS). Based on the sample size estimate, 15 preterm newborns hospitalized in the NICU who met the inclusion and exclusion criteria were selected on a consecutive basis.

For comparison purposes, we evaluated the salivary cortisol values of 15 term newborns, who were healthy and without comorbidities and hospitalized in the infant and mother ward of the same hospital.

This study was approved by the Research Ethics Committee of the State University of Rio de Janeiro under number 42594515.7.0000.5259, and a consent form was signed by the guardians of each study participant.

### 2.2. Intervention

**Figure 1:**
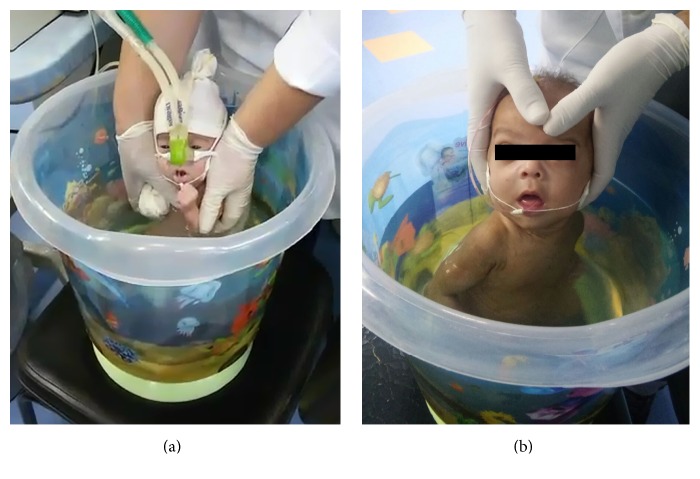
Infants undergoing hydrokinesiotherapy. Note the size of the plastic bucket (a) and the smooth movement of rotation (b).

After selection, the preterm infants were wrapped in a towel with the body semirelaxed and carefully placed in the liquid environment. To perform the neonatal hydrokinesiotherapy, a plastic bucket near the incubator was used. The bucket was filled with water up to the level of the newborn's shoulders and kept at a temperature of 37°C, monitored by a water thermometer (Optris LS LT, Berlin, Germany). This type of container resembles the shape of the uterus, allowing most of the newborn's body to remain submerged in a flexed and organized position [23]. The protocol lasted 10 minutes as the newborns performed light and slow movements aimed at tactile-kinesthetic stimulation and facilitating the flexed posture of body organization via the effect of the thrusting motion. The hydrokinesiotherapy comprised passive mobilizations of the upper and lower limbs, global stretching, trunk rotation, and tactile, proprioceptive, and vestibular stimulation, finishing with the baby in the fetal position [18]. At the end of the procedure, the postural organization was maintained, and the infant was wrapped in a towel and placed in an incubator (Table 1 and Figure 1). Physiological data were collected 5 minutes before and immediately after the intervention.

The physical therapists responsible for applying the technique (Welcy Cassiano de Oliveira Tobinaga and Cirlene de Lima Marinho) are both neonatology specialists and undertook an aquatic physical therapy protocol and evaluation scale training over a period of 15 days.

### 2.3. Data Collected

To minimize other potential stressors, all samples were obtained between 7:00 and 9:00 am because this time is considered to represent the peak cortisol level threshold, as well as being a time when less handling of the newborn, lower activity in the unit, and, consequently, lower noise and light exposure occur [11–14]. Physiological parameters were evaluated before and after neonatal hydrokinesiotherapy to estimate the pain level shown by the newborns included in the study. Respiratory rate, heart rate, peripheral capillary oxygen saturation (SpO_2_), and salivary cortisol values were recorded. Saliva samples were obtained using neutral Salivette® tubes (Landskrona, Sweden). The cotton swab was placed in the buccal mucosa of the infant for 3 min. After this period, the cotton swab was removed from the infant's mouth, and it was returned to the device. A saliva volume of 0.5 mL was considered sufficient for the test [11–14]. Pain levels were evaluated using the NIPS. This scale consists of the following indicators: facial expression (0 points: relaxed muscles; 1 point: grimace), crying (0 points: no crying; 1 point: whimpering; 2 points: vigorous crying), breathing pattern (0 points: relaxed; 1 point: different from baseline), position of the arms (0 points: relaxed; 1 point: flexed/extended), position of the legs (0 points: relaxed; 1 point: flexed/extended), and state of arousal (0 points: sleeping/quiet; 1 point: fussy). The maximum score is 7, and pain is considered to be present when the value is ≥4 points [26]. The NIPS has been previously adapted and validated for use in Brazil [27].

### 2.4. Statistical Analysis

To estimate the sample size, a pilot study in a group of eight preterm newborns was conducted using a protocol identical to that described above. Based on these preliminary results, MedCalc 8.2 software (MedCalc Software, Mariakerke, Belgium) was used to calculate the sample size based on the average values of salivary cortisol before and after the application of hydrokinesiotherapy, assuming a type I error of 5% and a type II error of 20% [28]. The minimal calculated value for this study consisted of 15 preterm newborns.

The Shapiro-Wilk normality test was used to evaluate the data distribution. Changes in the variables between the two time points (before and after hydrokinesiotherapy) were evaluated using a paired sample *t*-test or a Wilcoxon signed-rank test. Cortisol levels were compared using the paired sample *t*-test or Wilcoxon signed-rank test, depending on whether the groups were independent. The results are expressed as the mean ± standard deviation or as the frequency (percent). The analyses were performed using the software program Origin Pro 9.0 (OriginLab Corporation, Northampton, MA, USA). The level of statistical significance was *p* < 0.05.

## 3. Results

**Figure 2:**
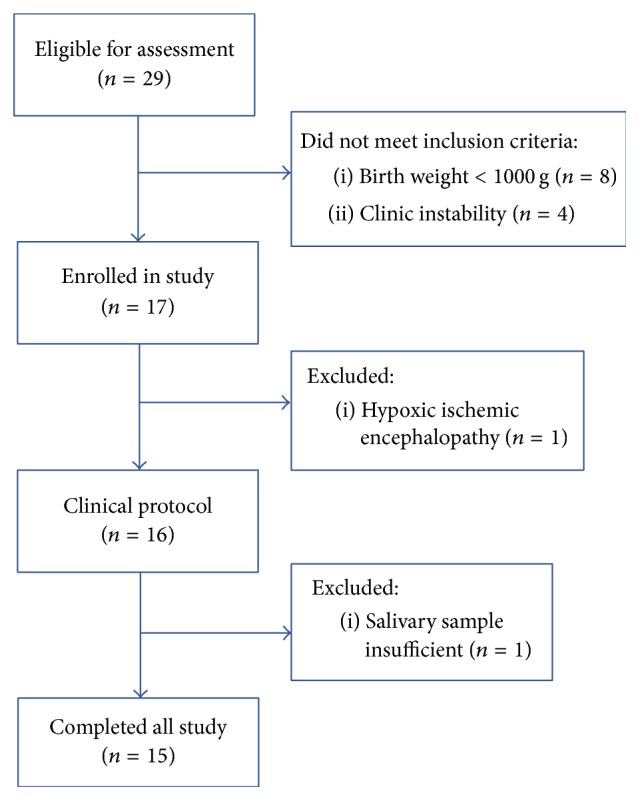
Flowchart showing the different stages of the recruitment process.

Of the 29 participants eligible for evaluation, 15 completed the study (Figure 2). The newborns had a mean gestational age of 34.2 ± 1.66 weeks and a mean weight of 1823.3 ± 437.4 g.

**Figure 3:**
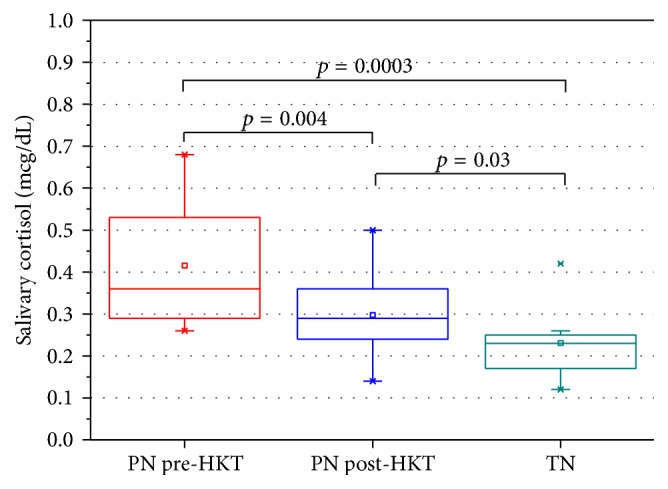
Comparison of salivary cortisol measurements between preterm newborns before (pre-HKT PN) and after (post-HKT PN) hydrokinesiotherapy and term newborns (TN). Significant differences were observed in salivary cortisol levels between the pre-HKT PN and post-HKT PN groups (*p* = 0.004). Significant differences were also found in salivary cortisol levels between the pre-HKT PN and TN groups (*p* = 0.0003) and between the post-HKT PN and TN groups (*p* = 0.03).

Before neonatal hydrokinesiotherapy, the preterm newborns had a mean salivary cortisol level of 0.41 ± 0.14 *μ*g/dL. After the procedure, the level was reduced to 0.29 ± 0.09 *μ*g/dL (*p* = 0.004). The mean salivary cortisol level of the term newborns was 0.23 ± 0.08 *μ*g/dL. A comparison of the salivary cortisol levels before and after hydrokinesiotherapy in preterm and term newborns is shown in Figure 3.

Regarding hemodynamics and respiratory parameters, the mean heart rate was 163.4 ± 14.1 beats/min before hydrokinesiotherapy and 150.4 ± 8.11 beats/min after the intervention, a difference that represented a significant reduction (*p* = 0.003). The mean respiratory rate was 55.2 ± 9.16 breaths/min before the procedure and 49.3 ± 7.90 breaths/min after the intervention, a difference that represented a significant reduction (*p* = 0.004). The mean SpO_2_ was 97 ± 2.64% before the procedure and 99 ± 1.05% after the intervention, a difference that represented a significant increase (*p* = 0.002). The mean axillary temperature was 36.4 ± 0.21°C before the procedure and 36.6 ± 0.35°C after the intervention, with no significant difference (*p* = 0.125). Regarding pain evaluation, the mean NIPS score before the procedure was 0.53 ± 0.83. After the procedure, the score for all patients was zero. However, this change was not significant (*p* > 0.05) (Table 2).

## 4. Discussion

Because of its goal of preventing and treating various clinical conditions, hydrokinesiotherapy is considered a neonatal therapeutic modality and has been shown to be beneficial in clinically stable newborns [21–23]. Several studies have shown that hydrokinesiotherapy helps to reduce pain, stress, irritability, and neuromusculoskeletal changes acquired by newborns during long periods of hospitalization [3, 9, 18, 19, 23–25]. In clinical practice in the NICU, hydrokinesiotherapy has been used on newborns with movement/tone abnormalities, developmental changes like lethargy and irritability, noninfectious respiratory system changes, and feeding difficulties, among other indications [12, 18, 25, 29]. Another potential application for hydrokinesiotherapy is infants with neonatal abstinence syndrome during the drug withdrawal phase in which sleep disruption and jittery movement are present [30]. It is important to note that safety aspects of hydrokinesiotherapy are still a problem in regard to its use with preterm newborns, due to the risks of worsening any acute infectious/inflammatory processes, abrupt changes to body temperature, worsening of neurological or cardiovascular conditions, and worsening of the base clinical condition, among other considerations [18, 19]. In the present study, we excluded newborns of extremely low weight (<1000 g) because hydrokinesiotherapy complications occur more often in these infants due to their greater difficulty in maintaining body temperature, which in turn may be due to immaturity of the central nervous system, thinness of the subcutaneous cellular tissue, a lower tissue oxygen supply, and a greater surface-mass ratio, all of which inevitably cause greater heat dissipation [31]. Moreover, we adopted as exclusion criteria the presence of intravascular cannulas due to the increased risk of infection and extravasation injuries by capillary fragility [32, 33].

In this study, preterm newborns showed a significant reduction in both heart rate and respiratory rate after neonatal hydrokinesiotherapy. Sweeney [23] observed decreased heart rates after hydrotherapy, suggesting that this change is associated with the shift to a behavioral state of comfort and relaxation provided by the physical properties of the water. Harrison et al. [34] evaluated 42 preterm newborns when they were not being handled or disturbed and observed that tachypnea may occur as a result of stress or pain and that important relationships exist between physiological stress, behavioral stress, and motor activity signals in preterm infants. Despite the fact that statistically significant reductions in heart and respiratory rates are probably not relevant from a clinical point of view, it is difficult to determine the real impact of these changes on preterm newborns when their physiological and behavioral systems are severely hyperstimulated compared with exposure to intrauterine stimuli [35]. It is also important to note that although the absolute values were within the limits of normality, the reductions in heart and respiratory rate strongly support the belief that hydrotherapy does not cause stress or pain.

In our study, no significant difference was observed in the temperature of the preterm newborns before and after application of the protocol. Our findings are in line with those of Sweeney [23], who evaluated the effects of hydrotherapy on term newborns and found that their temperature maintained a pattern within the bounds of normality. If the water temperature is close to that of the neonates, the conduction and convection mechanisms do not have an effect on heat loss. These two mechanisms are important for the dissipation of heat in aquatic exercise due to the large body surface area in contact with the water and the environment's conduction capacity [36].

Pulse oximetry is the simplest way to monitor oxygenation and respiratory stability in preterm infants [37]. In the present study, we observed a significant increase in SpO_2_ values after application of neonatal hydrokinesiotherapy (*p* = 0.002). Similar effects were found by Barbosa [19], who studied preterm newborns that were agitated and crying prior to therapy, with a mean SpO_2_ of 94.3 ± 1.70. After the technique, a significant increase was observed in SpO_2_ values (97.5 ± 0.80; *p* = 0.0002). Interestingly, Harrison et al. [34] reported that low SpO_2_ values are critical indicators of physiological stress. Among the many therapeutic effects of water, improved blood circulation caused by the effect of hydrostatic pressure, which mainly increases blood flow in the alveoli, may at least partly explain the improvement in gas exchange [38]. In our study, it should be noted that, despite the statistically significant difference between SpO_2_ values before and after hydrokinesiotherapy, the clinical relevance is low because the values all fall within the bounds of normality. However, the respiratory and neurological systems of preterm newborns are exposed to various complications and can never be compared with those of term newborns [37].

Until the 1950s, it was believed that newborns were unable to feel pain, and many professionals were not concerned about this issue, citing neurological immaturity of the connections responsible for the pain sensation, which possibly increases the pain threshold [3]. Neonates are often subjected to stimuli of a painful and more diverse nature within the NICU, and premature neonates are even more sensitive to pain and stress than term neonates [39]. Pain evaluation scales are important tools that can be applied before, during, and after a painful stimulus. NIPS is a newborn pain evaluation scale that is easy to understand and apply clinically which can help newborn healthcare professionals in situations where they wish to evaluate pain or pain relief [40]. Given that hydrokinesiotherapy is a technique that has rarely been studied in the literature, it would be opportune to evaluate this therapy using NIPS in two respects: from the point of view of hydrokinesiotherapy as both a procedure that can potentially cause pain and, contrariwise, a technique that can relieve pain and stress in the preterm newborn who is subject to constantly repeated or prolonged stimuli within the NICU [18, 19]. Despite the fact that the NIPS evaluation of pain perception did not reveal a significant difference, the clinical relevance of our study cannot be disregarded, as a score of zero was observed in all newborns after the intervention, demonstrating an absence of pain and adverse responses to the procedure. Findings similar to ours were obtained by Barbosa [19] in a study of 10 preterm newborns where hydrotherapy was performed for 10 minutes in the form of passive mobilization of the upper and lower limbs, global stretching, trunk rotation, and tactile-proprioceptive-vestibular stimulation. They noted that the NIPS scores remained below three after hydrotherapy (mean 0.3 ± 0.8), suggesting an increased pain threshold after the procedure. Interestingly, these authors also observed that NIPS scores increased from 3.5 to 4 when evaluating the control group without hydrotherapy, suggesting that the environment in which the preterm newborns were situated somehow provoked stress or pain. Vignochi et al. [18] selected 12 clinically stable neonates who had behavioral abnormalities such as intolerance to touch, excessive crying, difficulty in the transition from the crying phase to deep sleep, and pain signs according to the Neonatal Facial Coding System (NFCS) scale. Their results showed that the pain evaluation scale score decreased from 5.38 ± 0.91 to 0.25 ± 0.46  (*p* < 0.001) after application of aquatic physiotherapy. These results led the authors to suggest that aquatic physiotherapy is also a helpful resource for improving the sleep quality of these infants.

The present study revealed a significant reduction in salivary cortisol levels after hydrokinesiotherapy (*p* = 0.004). Cândia et al. [13] measured salivary cortisol levels in preterm newborns subjected to a prone position. Similar results were obtained with regard to a significant reduction in salivary cortisol levels (0.20 versus 0.13 *μ*g/dL; *p* = 0.003). This shows that positions and techniques that facilitate lower energy consumption, improved sleep quality, and better postural organization promote reductions in the potential harmful effects of hospitalization in the NICU [13]. According to Bates and Hanson [41], these beneficial results can be explained by the physiological effects of water on pain relief, acting at the level of the dorsal horn of the spinal cord by stimulating the major afferent receptors and conveying less painful messages. The physical properties of water may therefore be used to relieve pain in newborns. It is interesting to note that the circadian cycle of preterm infants is not coordinated with cortisol; this is possibly due to the immaturity of the central nervous system and the afferent and efferent suprachiasmatic nucleus pathways [14]. However, the circadian cycle is established between 2 and 16 weeks of life, in both premature and term newborns. The circadian cycle of adrenocorticotropic hormone release is related to an increase in its secretion in the morning period, between 5 am and 9 am, and a decrease during the nocturnal period [14]. As the duration of our protocol (including the two data collection periods) did not exceed 20 minutes and was always performed between 7 am and 9 am, we believe that the decrease in salivary cortisol after hydrokinesiotherapy could not have been due to the circadian cycle of this hormone.

When comparing the salivary cortisol levels in preterm newborns before and after hydrokinesiotherapy and with term newborns from the mother and infant ward, a significant difference was observed between the mean hormone levels. However, we found that, after hydrokinesiotherapy, the cortisol levels of infants in the NICU were closer to those of the term newborns in the ward. These results corroborate previous studies that have shown lower expression of behavioral signs indicative of stress after preterm newborns in the NICU underwent neonatal hydrokinesiotherapy [18, 19]. Interestingly, Cabral et al. [12] compared the stress responses of a group of newborns in the NICU (experimental group) to those of a group of healthy infants at home (control group) and found that the mean salivary cortisol levels on the 2nd and 9th day of life in the experimental group were 4.31 ± 2.65 *μ*g/dL and 1.83 ± 1.22 *μ*g/dL, respectively. The mean cortisol level observed in the control group on the 14th day of life (in the infant's home) was 1.02 ± 0.83 *μ*g/dL. According to these authors, the findings indicate an adrenal stress response during the first days of hospitalization, which may be related to suppression of the adrenal glands and interference in the stress response caused by the glucocorticoids that are often used during the prenatal period [12].

Similar to any other study, this work has its limitations. First, the presence of an age-matched control group with salivary cortisol measurements taken at the same times of day could have provided more conclusive information regarding levels of physical and psychological stress. Second, the sample size is relatively small, although this is explained by the fact that only preterm newborns were included. Third, only the short-term effects of neonatal hydrokinesiotherapy were evaluated. Despite these limitations, we believe that the results provide an important contribution to the literature, as no previous study has evaluated cortisol changes using hydrokinesiotherapy in preterm newborns. Future controlled trials with a larger number of infants that are conducted over a longer period could provide clearer information about the benefits provided by the application of hydrokinesiotherapy in the neonatal environment. This may affect the incorporation of this technique as routine therapy in the NICU.

In conclusion, neonatal hydrotherapy promoted short-term relief from feelings of stress in the present study. Neonatal hydrokinesiotherapy may be a therapeutic alternative in preterm newborns that is technically easy to administer and of low cost. However, it needs to be studied in randomized, crossover, and blinded trials.

## Figures and Tables

**Table 1:** Hydrokinesiotherapy protocol used in this study.

Steps	Procedures
One	Fill the bucket with sufficient water to promote immersion up to shoulder level with flotation of the newborn.
Two	Adjust the water temperature to 37°C and monitor the temperature using an aquatic thermometer.
Three	Remove the newborn from the incubator and transfer them to the bucket in a bent posture organized around the midline, with pulse oximetry monitoring of the upper right limb.
Four	Ideal: 2 caregivers: one to execute the technique and the other to help with the procedure if necessary.
Five	Gently immerse the newborn in the bucket with the water level up to his/her shoulders.
Six	Perform subtle pelvic dissociations with anteroposterior, laterolateral, and superoinferior trunk movements, using the physical properties of the water, such as buoyancy and flotation. Agitated and crying newborns may benefit from physical contact with the sides of the bucket, simulating intrauterine containment.
Seven	Stimulate active movement of the upper and lower limbs.
Eight	Facilitate the newborn's trunk rotation, both to the right and to left, using subtle movements with the aid of flotation.
Nine	Promote tactile-kinesthetic stimulation using light and slow movements, sliding the newborn in the liquid environment.
Ten	Repeat the maneuvers described above, and maintain postural organization. Close the protocol after 10 minutes with the newborn in the fetal position.
Eleven	After hydrokinesiotherapy is complete, return the newborn to the bed in the same form in which he/she was found before the intervention.

**Table 2:** Physiological values measured 5 minutes before and immediately after the intervention.

Parameter	Before hydrokinesiotherapy		After hydrokinesiotherapy		*p* value
	Mean ± standard deviation	Range	Mean ± standard deviation	Range	
Heart rate (beats/min)	163.4 ± 14.1	133–186	150.4 ± 8.11	133–162	0.003
Respiratory rate (breaths/min)	55.2 ± 9.16	42–68	49.3 ± 7.90	38–68	0.004
SpO_2_ (%)	97 ± 2.64	92–100	99 ± 1.05	97–100	0.002
Axillary temperature (°C)	36.4 ± 0.21	36.2–36.6	36.6 ± 0.35	36.3–36.9	0.125
NIPS score	0.53 ± 0.83	0–2	0	0	0.087

NIPS: Neonatal Infant Pain Scale.
